# Pituitary Apoplexy as a Complication of COVID-19 Infection

**DOI:** 10.7759/cureus.43524

**Published:** 2023-08-15

**Authors:** Hassan Hussain, KVC Janaka, DT Muthukuda, SG Perera

**Affiliations:** 1 General Medicine, Sri Jayewardenepura General Hospital, Colombo, LKA; 2 Internal Medicine, Sri Jayewardenepura General Hospital, Colombo, LKA; 3 Endocrinology, Sri Jayewardenepura General Hospital, Colombo, LKA; 4 Pulmonology, Sri Jayewardenepura General Hospital, Colombo, LKA

**Keywords:** ground-glass opacities, covid-19, thrombocytopenia, sellar lesion, pneumonia, headache, pituitary macroadenoma, pituitary apoplexy

## Abstract

Pituitary apoplexy (PA) is a complication occurring due to hemorrhage and/or infarction in a pituitary adenoma due to various pathophysiological mechanisms. Herein, we report a case of a 47-year-old previously healthy male who presented with fever and reduced level of consciousness for one day. During the hospital stay, he was diagnosed with PA in a background of pituitary macroadenoma along with positive nasopharyngeal swabs for SARS-CoV-2 infection. Even though the PA was successfully managed, the patient succumbed four days after admission due to respiratory failure caused by severe COVID-19 pneumonia.

## Introduction

Even though respiratory pathology is the hallmark of COVID-19, it can also cause numerous extrapulmonary manifestations [1]. Pituitary apoplexy (PA) is a rare life-threatening extrapulmonary manifestation, reported in patients with COVID-19 infection and pituitary adenoma [2]. PA is usually characterized by severe headache, visual disturbances, cranial nerve pathologies, and hormonal imbalances [3]. It has a very high mortality if not managed properly. In the presence of COVID-19, pneumonia risk is further increased.

Here, we report a case of a patient with a diagnosed COVID-19 infection and who was lately diagnosed with PA. It is probable that a possible association between the two entities led to the development of PA as reported.

## Case presentation

A 47-year-old healthy male presented with sudden onset fever and reduced level of consciousness for one-day duration. He had a history of intermittent non-specific headache for the past two years without red flag symptoms (early morning headache, visual disturbances, and vomiting), to which no medical advice was taken.

He also had a history of excessive sleepiness and lethargy for the same duration with no identifiable change in weight, appetite, or libido. He is a non-smoker and a teetotaler, and there was no family history of multiple endocrine neoplastic syndrome (MENS) or any other malignancies.

On admission, he was drowsy. He had a Glasgow Coma Scale (GCS) of 11/15 (eye 4, verbal 2, and motor 5), and no focal neurological signs or evidence of meningeal irritation was noted. He was febrile (38.3°C), with a pulse rate of 122 beats per minute and blood pressure of 90/60 mmHg. Respiratory rate was 20 breaths per minute with bilateral equal air entry and bibasal coarse crepitations. The rest of the cardiovascular, abdominal, and neurological systemic examinations were normal. 

His investigations on admission revealed a normal white cell count of 9.09 × 103/ µL, neutrophil 62% with a mild eosinophilia (2.6%), and thrombocytopenia (platelet count 90,000/µL). C-reactive protein (CRP) level was 29 mg/dl with a sodium level of 152 mEq/L, potassium was 3.2 mEq/L, and random plasma glucose was 110 mg/dl. His baseline troponin I was 1.25 ng/ml, rising to 25 ng/ml with dynamic electrocardiographic (ECG) changes. Serum ionized calcium and magnesium levels were within normal limits. Urine osmolality was 755 mOsmol/L, while serum osmolality was 318 mOsmol/L. Clotting profile and blood picture were normal. Investigations that were done are presented in Table 1.

**Table 1:** Investigations performed on the patient PT/INR: prothrombin time/international normalized ratio; VDRL: venereal disease research laboratory; TPHA: treponema pallidum hemagglutination; IgM: immunoglobulin M; IgG: immunoglobulin G; HIV: human immunodeficiency virus

		Day 1	Day 2	Day 3	Day 4	Normal range
Full blood count	Hemoglobin (g/dL)	11.4	11.6	10.4	8.3	11-13
	White cells (× 109 /L)	9.09	11.96	7.87	8.68	4.5-11
	Platelets (× 109/L)	98	124	82	104	150-400
Serum electrolytes	Serum potassium (mmol/L)	3.2	3.5	3.1	3.3	3.5-5.5
	Serum sodium (mmol/L)	134.5	144.6	151.3	150	135-145
	Serum creatinine (umol/L)	125	105	103	109	<100
	Serum corrected calcium (mg/dL)				9.7	8.5-10.2
	Serum magnesium (mmol/L)				0.9	0.85-1.10
Liver biochemistry	Aspartate transaminase (U/L)		276.2	163	109	<40
	Alanine transaminase (U/L)		108.8	78.4	67	<40
	Alkaline phosphatase (U/L)		51	35.7		<120
	Total bilirubin (mg/dL)		1.1	0.8	1.6	0.30-1.20
Clotting profile	PT/INR (international normalized ratio)	1.36			1.32	<1
	Activated partial thromboplastin time (sec)		31.9		38.4	
Infection screening	C-reactive protein (mg/L)	29	82	41	81	<5
	Urine full report	Normal				
	Urine culture	Negative				
	Serum VDRL and TPHA	Non-reactive				
	HIV 1 and 2 antibodies	Non-reactive				
	Dengue antigen	Negative				
	Dengue antibody IgM	Negative				
	Dengue antibody IgG	Positive				
Other blood investigations	Serum osmolality (mOsmol/L)	318				
	Blood urea (mg/dl)	39	38	50	54	15-40
Urine osmolality (mOsmol/L)			755			
Cardiac biomarkers	Troponin I (ng/ml)	1.250		24.454	23.071	<0.034
	Pro brain natriuretic peptide (pro BNP) (pg/ml)	5219				<349
Hormonal profile	Prolactin (ng/ml)			18.01		<15
	Thyroid-stimulating hormone (TSH) (µIU/ml)		1.093			0.35-4.94
	Free triiodothyronine (fT3) (pg/ml)		1.57			2-4.40
	Free thyroxine (fT4) (ng/dl)		0.65			0.93-1.70
	Serum cortisol (nmol/L)	220.9				73.8-291

Regarding his hormonal profile, serum cortisol was in the upper limit of normal (220.9), with normal thyroid-stimulating hormone (TSH) levels and reduced triiodothyronine (T3) and thyroxine (T4) levels. His prolactin level was slightly elevated.

Because of ongoing fever, infection screening was performed, including dengue antigen and antibody, venereal disease research laboratory test (VDRL) value, human immunodeficiency virus 1 (HIV) 1 and 2 antibody, and hepatitis B surface antigen, which were all found to be negative.

**Figure 1:**
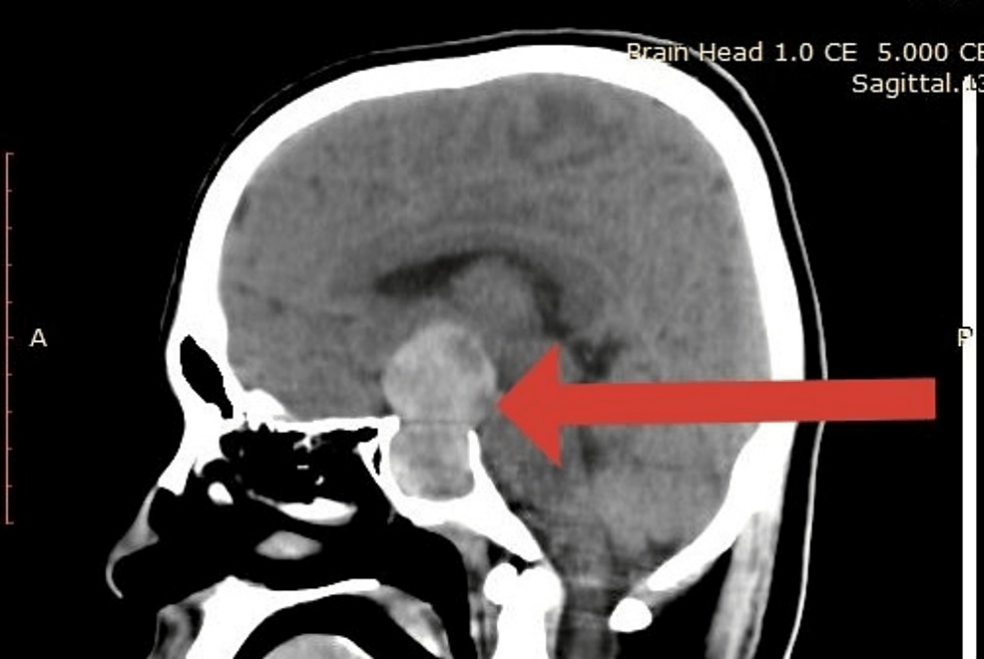
Non-contrast computed tomography (NCCT) brain sagittal view demonstrating a hyperdense large sellar lesion with hypodensity within the hyperdense area seen. Suprasellar and parasellar extensions were also noted. Indicates a possible pituitary macro adenoma with bleeding into the tumor.

Based on the clinical findings, presumptive diagnosis of meningoencephalitis was made, and he was initiated on empirical intravenous (IV) ceftriaxone along with efforts to maintain the hemodynamic stability with IV fluid boluses. An urgent non-contrast computed tomography (NCCT) brain was done, which demonstrated a hyperdense large sellar lesion with hypodensity within. Suprasellar and parasellar extensions were also noted. It was indicative of pituitary macroadenoma with bleeding into the tumor (Figure 1). The patient was diagnosed with PA and was started on IV hydrocortisone and vasopressors to maintain the mean arterial pressure (MAP) above 65.

A multidisciplinary team approach, including endocrine and neurosurgical opinion, was taken. As the GCS started to drop, the patient was transferred to the intensive care unit (ICU) for mechanical ventilatory support. Rapid antigen test for COVID-19 was performed, which turned to be positive.

On day 2 of admission, his GCS improved up to 14/15, but lung signs deteriorated progressively, requiring further ventilatory support. Chest X-ray showed pan lobar pattern of lung involvement with multifocal airspace opacities (ground-glass opacities and consolidation) suggestive of COVID-19 pneumonia. Hence, he was transferred to a dedicated COVID-19 ICU. Even though PA was managed, two days later, the patient unfortunately passed away due to complications of severe COVID-19 pneumonia.

## Discussion

The SARS-CoV-2 virus causes a complex infectious disease with a diverse clinical presentation and complications [1]. Identifying possible consequences of the said viral infection had been challenging throughout due to its multisystemic involvement. Even though cardiac surgery, head trauma, and anticoagulation treatment have shown to precipitate PA, in more than 50% of cases, no predisposing factor was identified [2,4].

Among the postulated hypotheses for the possible association of COVID-19 and PA, marked inflammatory response associated with COVID-19 leading to vascular dysfunction, which in turn leads to PA, is identified as a major pathophysiological mechanism [2,5]. However, this relationship could not justify the development of PA in some COVID-19 patients. Another mechanism is angiotensin-converting enzyme 2 (ACE-2) receptor-associated direct cell death. It was found to be a more plausible mechanism as these ACE-2 receptors are found in abundance in pituitary cells [2,6]. SARS-CoV-2 infection was found to have an effect on the clotting cascade by inducing thrombocytopenia and dysfunction of platelets, thereby precipitating pituitary hemorrhage [7].

The striking feature in our patient was altered level of consciousness, which is usually seen in around 20% of PA patients [4]. This is possibly due to increased intracranial pressure [4]. Majority (80%) of patients with PA present with sudden onset severe frontal or retroorbital headache due to meningeal irritation secondary to the enlargement of the sella turcica [4]. The history of recurrent headaches in our patient for two years could have been a sign of pituitary macroadenoma. Since it was confined to the sella, the headache might not have been severe enough for him to seek medical advice for the past two years [3]. In more than 50% of patients, visual disturbances become a striking feature due to compression of the optic chiasm, but it was not seen in our patient, possibly due to the fact that minor visual changes are attributed to aging by the majority, even if those changes are present.

Imaging plays a major role in the management of a patient with a suspected intracranial and respiratory pathology. NCCT brain and chest radiograph were used in our patient, which showed features of PA and COVID-19 pneumonia, respectively. Ideal investigation to diagnose PA would have been magnetic resonance imaging (MRI) brain, which was not available as the first-line investigation in our case [2]. In an acute presentation, NCCT brain and clinical presentation were adequate for the accurate diagnosis to exclude other acute cerebrovascular events [2,4]. Considering the hormonal profile, hypocortisolism is usually seen in the initial stages of PA [4]. This patient also had serum cortisol in the lower limit of normal, which is inappropriate and significantly low for a patient in shock. Mild hypothyroidism was also seen in our patient, even though it usually occurs over weeks if its due to PA [4].

Managing PA includes hemodynamic stabilization, correction of electrolyte imbalances, and administration of corticosteroids to counteract the hormonal deficiencies and to reduce cerebral oedema [4]. This was appropriately managed in our patient, but still due to severe COVID-19 pneumonia, he succumbed to his illness four days after the diagnosis of PA.

## Conclusions

The association between PA and COVID-19 is not just a mere coincidence, but a result of COVID-19 infection inducing well-known precipitants of PA by a number of mechanisms, which are still not well understood. Hence, the possibility of COVID-19 infection should also be considered in patients diagnosed with PA and vice versa, i.e., in a COVID-19-infected patient presenting with features of shock and low GCS or headache. A timely diagnosis, investigations, and management are paramount for a better outcome.
